# 辅助化疗与靶向治疗在I期肺腺癌术后患者中应用的研究进展

**DOI:** 10.3779/j.issn.1009-3419.2024.101.26

**Published:** 2024-10-20

**Authors:** Ke ZHAO, Chao GUO, Yeye CHEN, Shanqing LI

**Affiliations:** 100730 北京，中国医学科学院北京协和医院胸外科; Department of Thoracic Surgery, Peking Union Medical College Hospital, Chinese Academy of Medical Science & Peking Union Medical College, Beijing 100730, China

**Keywords:** 辅助治疗, 化疗, 靶向治疗, I期, 肺腺癌, Adjuvant therapy, Chemotherapy, Targeted therapy, Stage I, Lung adenocarcinoma

## Abstract

肺癌是我国癌症负担和癌症死亡的主要原因之一，每年有近80万人新诊断为肺癌患者，其中接近一半的患者为肺腺癌（lung adenocarcinoma, LUAD）。根据目前的临床指南，手术是I期LUAD患者的主要治疗手段，但是仅靠手术I期LUAD患者的5年生存率仍不令人满意，为73%-90%，即有相当一部分患者需要其他手段来提高生存获益。化疗和靶向治疗已经在局部晚期和转移性LUAD患者的治疗中取得了巨大的成就，但是能否使I期LUAD术后患者获益仍存在争议。基于该现状，已经有很多研究者关注到了这一问题并做出了有益的探索。本综述就影响I期LUAD术后患者接受辅助化疗和靶向治疗的因素以及辅助化疗和靶向治疗在I期LUAD术后患者中应用的相关临床研究作了简单的回顾，以期更广泛地了解该问题的最新进展并为推动该领域的持续研究寻找新的突破点。

## Abstract (en)

Lung cancer is one of the main causes of cancer burden and death in China, with nearly 800,000 newly diagnosed lung cancer patients each year, nearly half of whom are lung adenocarcinoma (LUAD) patients. According to current clinical guidelines, surgery is the main treatment for stage I LUAD patients, but the 5-year overall survival rate of stage I LUAD patients alone is still unsatisfactory, about 73%-90%, indicating that a considerable number of patients require other means to improve survival benefits. Chemotherapy and targeted therapy have achieved great success in the treatment of locally advanced and metastatic LUAD patients, but there is still controversy over whether they can benefit stage I LUAD postoperative patients. Under the circumstances, many researchers have paid attention to this issue and made beneficial explorations. This review provides a brief review of the factors that affect the acceptance of adjuvant chemotherapy and targeted therapy in stage I LUAD postoperative patients, as well as the relevant clinical research on the application of adjuvant chemotherapy and targeted therapy in stage I LUAD postoperative patients, in order to gain a broader understanding of the latest developments in this field and find new breakthroughs to promote sustained research in this field.

肺癌是一组起源于支气管上皮组织的恶性肿瘤，其中肺腺癌（lung adenocarcinoma, LUAD）是目前临床上最常见的类型。现在通常认为LUAD是一组具有遗传和细胞异质性的独特疾病^[1,2]^。由于烟草滥用、空气污染、放射性暴露以及慢性肺部疾病等原因，我国肺癌的发病率和死亡率一直居高不下^[3]^。

针对LUAD的治疗，要根据肿瘤原发灶-淋巴结-转移（tumor-node-metastasis, TNM）分期系统（如无特殊指出，本文提到的TNM分期指第八版）来做出最佳选择^[4]^，对于符合条件的患者来说，手术是治疗I-IIIA期LUAD的关键因素。但即使接受了手术，也会有10%-27%的I期患者可能在确诊后5年内去世^[4]^。以铂类药物为基础的辅助化疗（adjuvant chemotherapy, ACT）在II期和部分III期LUAD患者中的作用已经得到公认^[5,6]^，辅助靶向治疗（adjuvant targeted therapy, ATT）也在相应分期的患者中取得了令人瞩目的研究结果^[7,8]^，但是对于I期LUAD术后患者，是否需要接受ACT或ATT仍存在着很大的争议。本文拟梳理目前关于I期LUAD术后患者辅助治疗相关研究的进展。

## 1 影响LUAD患者辅助治疗疗效的因素

### 1.1 肿瘤病理因素

#### 1.1.1 肿瘤分化程度

肿瘤的分化程度是影响患者治疗选择的一个重要因素。2020年6月，国际肺癌研究协会（International Association for the Study of Lung Cancer, IASLC）提出了浸润性LUAD的组织学分级系统^[9]^，该分级系统将浸润性LUAD分为了低级别、中级别和高级别三类，多项研究^[10⇓-12]^证实，随着级别的升高，浸润性LUAD的预后依次变差。在高级别组中，ACT对IB-III期的患者有显著的总生存期（overall survival, OS）获益（P=0.048），多变量分析进一步证实，ACT是高级别组肿瘤OS的独立预后因素（P=0.002）^[12]^。但是靶向治疗对早期高级别浸润性LUAD患者的生存益处尚未见报道。上述研究说明，肿瘤分级系统将帮助我们筛选出能从辅助治疗中获益的早期肺癌患者。

#### 1.1.2 肿瘤高危病理因素

脉管瘤栓目前被认为是肺癌患者的不良预后因素^[13]^，在I期非小细胞肺癌（non-small cell lung cancer, NSCLC）患者中，合并脉管瘤栓的患者预后也更差^[14⇓-16]^。有研究^[17]^指出，这种预后意义在早期LUAD中体现得更为明显，因此应当有针对性地设计辅助治疗的前瞻性试验。

气腔播散也被认定为是LUAD的不良预后因素^[18,19]^。这一结论在早期患者中尚有争议，大多数研究^[20⇓-22]^支持合并气腔播散的I期LUAD患者预后更差，应当接受辅助治疗以减少复发转移，但是也有研究^[16]^指出在切除的早期LUAD中，气腔播散似乎不会影响患者的复发或死亡率。因此气腔播散是否能够作为影响患者术后辅助治疗的因素还需要更大规模更严谨的研究来提供证据。

### 1.2 临床因素

#### 1.2.1 影像学表现

胸部计算机断层扫描（computed tomography, CT）中的一些影像学特征比如胸膜侵犯、肿瘤最大径将直接影响患者的TNM分期从而影响患者预后^[4]^，但还有一些特点并未影响TNM分期，但是也有一定的预后意义。一部分肿瘤在胸部CT上表现为空洞样的改变，有研究^[23,24]^指出，空洞样的病灶比非空洞样的病灶预后要更差，是复发和总OS的独立危险因素。当然，也有反面的研究结果不支持这一结论，比如Nguyen等^[25]^的研究显示CT上的空洞表现无法预测患者的生存，但此研究的样本量较小，结论需要仔细推敲。

日本临床肿瘤研究组（Japan Clinical Oncology Group, JCOG）系列研究^[26⇓⇓-29]^的结论让我们认识到肿瘤在胸部CT上的实性成分占比（consolidation tumor ratio, CTR）也对治疗方式选择有着巨大的影响，有研究者探讨了CTR与预后的关系，结果提示更高的CTR预示着更差的预后^[30⇓-32]^。Park等^[31]^发现，纯实性的肿瘤更容易出现复发和远处转移，磨玻璃结节（ground-glass nodule, GGN）的存在与更晚的复发时间和更长的OS相关（P<0.001）。Wang等^[30]^的研究规模较大，纳入了2775例患者，其结果显示纯GGN组5年总OS率和无病生存（disease-free survival, DFS）率分别为98.8%和98.0%，部分实性组分别为96.0%和86.5%，纯实性组分别为88.0%和75.5%（P<0.001），说明目前TNM分期的局限性，也提示对于CT上表现为纯实性的肿瘤，辅助治疗可以适当积极一些。

正电子发射断层显像/CT（positron emission tomography/CT, PET/CT）越来越多地被应用到肺癌患者的常规诊疗当中^[33]^，也有多项研究^[34⇓⇓-37]^发现了PET/CT相关参数对NSCLC患者的预后意义。一项荟萃分析^[36]^汇集了36项研究，其结论为最大标准化摄取值（maximal standardized uptake value, SUVmax）、代谢肿瘤体积（metabolic tumor volume, MTV）和病变糖酵解总量（total lesion glycolysis, TLG）的高值预示着手术NSCLC患者的复发或死亡风险更高，这一结论在I期患者中也成立。Sharma等^[37]^的研究则是一项前瞻性研究，其主要结论为MTV和TLG是接受铂类化疗的NSCLC患者OS的可靠预后标志物。

#### 1.2.2 手术切除范围

虽然亚肺叶切除已经在某些特定人群中取得了成功^[26,27,29]^，但是仍有部分患者的亚肺叶切除是妥协性的，常见原因包括术中发现全胸膜腔黏连、冰冻肺门、患者预期肺功能较差等。在一项根据监测、流行病学和最终结果（Surveillance, Epidemiology, and End Results, SEER）数据库的研究中，研究者发现在≤2 cm的肿瘤中，肺叶切除术的5年OS率高于亚肺叶切除术^[38]^。这一结果受到了一些质疑，质疑点在于研究者没有平衡两组的基线特征，并且忽略了在亚肺叶切除中淋巴结被完全解剖的可能性较小这一问题^[39]^，但是后续有样本量更大的研究，在通过倾向性评分平衡组间混杂因素后还是支持肺叶切除术对I期肺癌患者预后的优势地位，并且说明辅助治疗能够改善亚肺叶切除组的预后^[40]^。

### 1.3 生物标志物

#### 1.3.1 影响ACT选择的生物标志物

既往有研究^[41⇓-43]^发现了一些可能影响NSCLC患者预后以及化疗获益的基因，包括切除修复交叉互补基因1（excision repair cross complementary gene 1, ERCC1）、核苷酸还原酶1（ribenucleotide reductase M1, RRM1）、乳腺癌易感基因1（breast cancer susceptibility gene 1, BRCA1）和p53抑癌基因，但这些研究结果并未在临床上得到广泛应用，主要还是存在样本处理不够标准化、检测方法不可靠及抗体的特异性差、信使核糖核酸和蛋白检验的异质性等问题。

目前比较有前景的方法是循环肿瘤DNA（circulating tumor DNA, ctDNA）指导早期NSCLC的干预和个性化辅助治疗。Qiu等^[44]^的研究指出，ctDNA可作为NSCLC术后和ACT后风险分层和早期检测复发的可靠生物标志物，并且能筛选从ACT中获益的优势人群。此外，肿瘤突变负荷（tumor mutational burden, TMB）也有一定的指导意义，Song等^[45]^的研究发现，低TMB的患者更能从化疗中获益，其无进展生存期（progression-free survival, PFS）明显长于高TMB的患者[风险比（hazard ratio, HR）=0.554, P=0.036]。

#### 1.3.2 影响ATT选择的生物标志物

作为精准医学的代表治疗方法，酪氨酸激酶抑制剂（tyrosine kinase inhibitor, TKI）等靶向治疗主要应用在LUAD患者当中。表皮生长因子受体（epidermal growth factor receptor, EGFR）、间变性淋巴瘤激酶（anaplastic lymphoma kinase, ALK）、c-ros肉瘤致癌因子1（ROS proto-oncogene 1, ROS1）、间质表皮转化因子（mesenchymal to epithelial transition factor, MET）以及RET等靶点本身就是预测靶向治疗效果的标志物，但针对这些靶点的大多数药物尚未在I期患者的辅助治疗中得到广泛的应用^[46⇓⇓-49]^。在此，我们简单介绍一些新兴的可能改变临床实践的靶点。

有研究^[50,51]^认为神经调节蛋白-1（neuregulin-1, NRG1）融合在浸润性肺黏液性腺癌中富集（约30%的黏液腺癌患者中存在NRG1突变）。已经发现泛ERBB抑制剂类药物（如阿法替尼）对浸润性肺黏液性腺癌的治疗具有潜在的敏感性^[52⇓-54]^。Kirsten大鼠肉瘤病毒癌基因同源物（Kirsten rat arcomaviral oncogene homolog, KRAS）突变见于高达20%-30%的NSCLC患者，是最常见的肺癌致癌驱动因素。虽然目前针对KRAS的药物尚停留在I期研究^[55,56]^，但由于它和许多其他主要驱动基因相互排斥，可用于排除样本进行其他可成药改变（如EGFR突变和ALK融合）的进一步检测^[57]^。

## 2 辅助治疗在I期LUAD术后患者中应用的相关临床研究

### 2.1 ACT在I期LUAD术后患者中应用的相关临床研究

最新版的美国国立综合癌症网络（National Comprehensive Cancer Network, NCCN）指南已经不再推荐对高危的IB期术后患者进行ACT^[58]^，这主要是因为既往的大规模随机对照研究（randomized controlled trial, RCT）均未得出辅助治疗对I期术后患者有利的结论，而以第八版TNM分期为基础的研究又非常少。我们也未检索到近期的针对I期LUAD术后患者辅助化疗的前瞻性RCT，仅有多篇回顾性研究提供了有争议的结论。

2013年一项韩国的回顾性研究^[59]^指出，对于肿瘤大小介于3-4 cm且没有淋巴结和远处转移的患者，铂类ACT是OS（HR=0.428, P=0.049）和DFS（HR=0.57, P=0.043）的良好预后因素，但是该研究的入组人群有偏倚，观察组患者的平均年龄较高，而治疗组患者的体能状态较好。Morgensztern等^[60]^的研究样本量更大，纳入10,587例IB期患者，其中接受ACT的有1608例，结果发现接受ACT的IB期患者的中位生存期较观察组延长了33.4个月（P<0.001）。

Tu等^[61]^的基于SEER数据库的研究纳入16,765例患者，其中LUAD患者6103例，ACT组的LUAD患者有1027例，经过倾向性评分匹配后的结果显示ACT组的OS优于非ACT组（HR=0.775, 95%CI: 0.704-0.853, P<0.0001），尤其是肿瘤大小在2 cm以上的患者。但这一研究纳入了肺段切除和肺楔形切除等手术方式，而且ACT组和非ACT组分别有约26.0%和26.7%的患者切除的淋巴结<4个，这些因素很可能导致一些分期更高的患者被纳入了研究，从而产生结论的偏倚。另一篇基于SEER数据库的研究^[62]^结论也支持ACT的生存益处，但是该研究在吸烟、手术并发症、淋巴血管浸润、体能状态和驱动基因突变等可能影响预后的因素上存在缺失。

Zhang等^[63]^的研究则得出相反的结论，在IB期NSCLC患者（超过80%的入组患者均为LUAD患者）中，ACT与5年OS率的改善没有显著相关性，仅仅在肿瘤大小介于3-4 cm之间且合并脏层胸膜受侵的亚组中才看到OS率的显著差异（93.98% vs 68.93%, P<0.01）。Cheng等^[64]^的研究也认为仅有在大小介于3-4 cm之间且为低分化肿瘤的亚组中，ACT才能带来更好的2年OS率。另一项重点针对IB期患者的研究^[65]^则是回顾了569例因原发性NSCLC接受肺切除术的患者，该研究发现对于IB期术后NSCLC患者，观察组的5年OS为87.6%，治疗组则为82.4%（P=0.021），ACT反而带来了更差的预后。

因此更多的研究将目标人群设定为高危的IB期术后患者，比如内脏胸膜受侵、脉管瘤栓、实性生长模式、微乳头生长模式以及淋巴结清扫数目较少（<16个），结果均提示ACT带来了生存益处^[66⇓⇓-69]^，但各项研究的入组标准均不统一。这些研究结论以及本文1.1和1.2中的内容提示我们，未来应当首先在合并高危因素的I期术后患者中开展RCT来明确ACT的治疗意义。

值得一提的是，Tsutani等^[69]^的研究还在IA3期的患者中发现了ACT的生存获益。这一结论与2010年进行的一项大型荟萃分析研究结论类似，该研究纳入了26项临床研究，结论为在手术的基础上加用化疗可使5年OS率从29%提高到33%，这种增加似乎不随患者分期而改变，即使是IA期患者也获得了2%的5年OS率提升^[70]^，虽然存活比例上升非常有限，但是由于肺癌患者数量巨大，ACT仍有可能为数万名患者带来生存获益，这说明未来ACT的治疗窗口仍有可能前移。

### 2.2 ATT在I期LUAD术后患者中应用的相关临床研究

在ATT方面，针对IB期术后患者的辅助治疗已经获得了重大突破。ADAURA研究^[7]^是一项III期的前瞻性RCT，目的是比较在肿瘤完全切除后，与安慰剂相比，辅助奥希替尼治疗EGFR突变的IB-IIIA期（第七版TNM分期）NSCLC（入组患者主要为LUAD患者）具有临床意义的DFS。在得出最终结果后，研究者对入组患者进行了重新分期，有199例患者被分为第八版的IB期，结果显示，奥希替尼组的5年DFS率为78%（95%CI: 67%-86%），安慰剂则只有55%（95%CI: 43%-65%）；DFS的HR为0.44（95%CI: 0.25-0.76）。该研究的次要结果还显示，奥希替尼可使研究全人群（IB-IIIA期患者）死亡风险相对下降51%，HR=0.49，5年OS率绝对值提高近10%（87.6% vs 77.7%），这让奥希替尼成为目前唯一在EGFR突变阳性NSCLC辅助治疗中达到显著OS获益的靶向药物。

ADAURA2研究^[71]^旨在探索奥希替尼对于接受完全切除的、复发高危的IA2、IA3期NSCLC患者（入组患者主要为LUAD患者）DFS的影响，目前研究尚在招募受试者中，主要终点的中期结果预计将于2027年8月公布。目前着眼于ATT在IA期LUAD术后患者中治疗意义的研究较为罕见。笔者最近发表的一项研究^[72]^发现，辅助靶向治疗可以显著降低合并高危病理因素的I期术后患者的复发风险（HR=0.34, 95%CI: 0.17-0.71, P=0.004），这一结论在IA2-IA3期亚组患者中同样成立。

CORIN研究^[73]^则是一项II期的开放标签随机研究，旨在评估完全切除的EGFR突变型IB期NSCLC患者（入组患者主要为LUAD患者）接受埃克替尼辅助治疗对DFS的影响，按照第八版TNM重新分期后，埃克替尼组的DFS显著长于观察组（HR=0.25, 95%CI: 0.07-0.87, P=0.018），此结果尚待后续III期RCT进一步验证。

对于合并ALK变异的LUAD患者，针对克唑替尼的ALCHEMIST研究和针对阿来替尼的ALINA研究^[74,75]^均未纳入第八版分期中I期的患者。Selpercatinib是一种首创、高选择性和强效的中枢神经系统活性RET抑制剂^[76]^，目前正在进行一项全球性、随机、双盲、III期试验的患者招募^[77]^，评估Selpercatinib与安慰剂在IB-IIIA期（第八版TNM分期）、RET融合阳性、接受根治手术的NSCLC患者中的疗效，其最终结果值得期待。其余靶点由于更为罕见，鲜有大规模的临床研究报道。

## 3 小结

目前关于I期LUAD术后的辅助治疗是否必要仍未能找到确定的答案，从目前的研究结果来看，合并EGFR敏感突变的IB期患者应当接受靶向治疗，而其他IB期患者（无论是否合并高危因素）应当首先被考虑进入到ACT的范围内，而对于IA3期和IA2期的患者，可以考虑对预期结局较差的患者尝试应用辅助治疗，但这需要谨慎的临床判断和与患者充分的讨论，以弥补前瞻性数据的空白。未来需要组织大规模的前瞻性RCT来阐明ACT和ATT对I期LUAD患者的真实意义。

## References

[1] Chen Z, Fillmore CM, Hammerman PS, et al. Non-small-cell lung cancers: a heterogeneous set of diseases. Nat Rev Cancer, 2014, 14(8): 535-546. doi: 10.1038/nrc3775 25056707 PMC5712844

[2] Relli V, Trerotola M, Guerra E, et al. Abandoning the notion of non-small cell lung cancer. Trends Mol Med, 2019, 25(7): 585-594. doi: 10.1016/j.molmed.2019.04.012 31155338

[3] Zheng RS, Chen R, Han BF, et al. Cancer incidence and mortality in China, 2022. Zhonghua Zhongliu Zazhi, 2024, 46(3): 221-231. 38468501 10.3760/cma.j.cn112152-20240119-00035

[4] Goldstraw P, Chansky K, Crowley J, et al. The IASLC lung cancer staging project: proposals for revision of the TNM stage groupings in the forthcoming (eighth) edition of the TNM classification for lung cancer. J Thorac Oncol, 2016, 11(1): 39-51. doi: 10.1016/j.jtho.2015.09.009 26762738

[5] Pignon JP, Tribodet H, Scagliotti GV, et al. Lung adjuvant cisplatin evaluation: a pooled analysis by the LACE Collaborative Group. J Clin Oncol, 2008, 26(21): 3552-3559. doi: 10.1200/JCO.2007.13.9030 18506026

[6] Bradbury P, Sivajohanathan D, Chan A, et al. Postoperative adjuvant systemic therapy in completely resected non-small-cell lung cancer: a systematic review. Clin Lung Cancer, 2017, 18(3): 259-273.e8. doi: 10.1016/j.cllc.2016.07.002 28162945

[7] Herbst RS, Wu YL, John T, et al. Adjuvant osimertinib for resected EGFR-mutated stage IB-IIIA non-small-cell lung cancer: updated results from the phase III randomized ADAURA trial. J Clin Oncol, 2023, 41(10): 1830-1840. doi: 10.1200/JCO.22.02186 36720083 PMC10082285

[8] He J, Su C, Liang W, et al. Icotinib versus chemotherapy as adjuvant treatment for stage II-IIIA EGFR-mutant non-small-cell lung cancer (EVIDENCE): a randomised, open-label, phase 3 trial. Lancet Respir Med, 2021, 9(9): 1021-1029. doi: 10.1016/S2213-2600(21)00134-X 34280355

[9] Moreira AL, Ocampo PSS, Xia Y, et al. A grading system for invasive pulmonary adenocarcinoma: a proposal from the International Association for the Study of Lung Cancer Pathology Committee. J Thorac Oncol, 2020, 15(10): 1599-1610. doi: 10.1016/j.jtho.2020.06.001 32562873 PMC8362286

[10] Rokutan-Kurata M, Yoshizawa A, Ueno K, et al. Validation study of the International Association for the Study of Lung Cancer Histologic Grading System of Invasive Lung Adenocarcinoma. J Thorac Oncol, 2021, 16(10): 1753-1758. doi: 10.1016/j.jtho.2021.04.008 33905897

[11] Fujikawa R, Muraoka Y, Kashima J, et al. Clinicopathologic and genotypic features of lung adenocarcinoma characterized by the International Association for the Study of Lung Cancer Grading System. J Thorac Oncol, 2022, 17(5): 700-707. doi: 10.1016/j.jtho.2022.02.005 35227909

[12] Deng C, Zheng Q, Zhang Y, et al. Validation of the Novel International Association for the Study of Lung Cancer Grading System for invasive pulmonary adenocarcinoma and association with common driver mutations. J Thorac Oncol, 2021, 16(10): 1684-1693. doi: 10.1016/j.jtho.2021.07.006 34302987

[13] Neri S, Yoshida J, Ishii G, et al. Prognostic impact of microscopic vessel invasion and visceral pleural invasion in non-small cell lung cancer: a retrospective analysis of 2657 patients. Ann Surg, 2014, 260(2): 383-388. doi: 10.1097/SLA.0000000000000617 24646539

[14] Shimada Y, Saji H, Yoshida K, et al. Pathological vascular invasion and tumor differentiation predict cancer recurrence in stage IA non-small-cell lung cancer after complete surgical resection. J Thorac Oncol, 2012, 7(8): 1263-1270. doi: 10.1097/JTO.0b013e31825cca6e 22673056

[15] Wang S, Zhang B, Qian J, et al. Proposal on incorporating lymphovascular invasion as a T-descriptor for stage I lung cancer. Lung Cancer, 2018, 125: 245-252. doi: 10.1016/j.lungcan.2018.09.024 30429028

[16] Nicotra S, Melan L, Pezzuto F, et al. Significance of spread through air spaces and vascular invasion in early-stage adenocarcinoma survival: a comprehensive clinicopathologic study of 427 patients for precision management. Am J Surg Pathol, 2024, 48(5): 605-614. doi: 10.1097/PAS.0000000000002199 38441164

[17] Suaiti L, Sullivan TB, Rieger-Christ KM, et al. Vascular invasion predicts recurrence in stage IA2-IB lung adenocarcinoma but not squamous cell carcinoma. Clin Lung Cancer, 2023, 24(3): e126-e133. doi: 10.1016/j.cllc.2022.12.006 36631388

[18] Shih AR, Mino-Kenudson M. Updates on spread through air spaces (STAS) in lung cancer. Histopathology, 2020, 77(2): 173-180. doi: 10.1111/his.14062 31943337

[19] Travis W D, Brambilla E, Nicholson A G, et al. The 2015 World Health Organization classification of lung tumors: impact of genetic, clinical and radiologic advances since the 2004 classification. J Thorac Oncol, 2015, 10(9): 1243-1260. doi: 10.1097/JTO.0000000000000630 26291008

[20] Chen Z, Wu X, Fang T, et al. Prognostic impact of tumor spread through air spaces for T2aN0 stage IB non-small cell lung cancer. Cancer Med, 2023, 12(14): 15246-15255. doi: 10.1002/cam4.6211 37278137 PMC10417161

[21] Kadota K, Nitadori JI, Sima CS, et al. Tumor spread through air spaces is an important pattern of invasion and impacts the frequency and location of recurrences after limited resection for small stage I lung adenocarcinomas. J Thorac Oncol, 2015, 10(5): 806-814. doi: 10.1097/JTO.0000000000000486 25629637 PMC4500042

[22] Huang L, Tang L, Dai L, et al. The prognostic significance of tumor spread through air space in stage I lung adenocarcinoma. Thorac Cancer, 2022, 13(7): 997-1005. doi: 10.1111/1759-7714.14348 35174646 PMC8977166

[23] Onn A, Choe DH, Herbst RS, et al. Tumor cavitation in stage I non-small cell lung cancer: epidermal growth factor receptor expression and prediction of poor outcome. Radiology, 2005, 237(1): 342-347. doi: 10.1148/radiol.2371041650 16183941

[24] Wang M, Zhao J, Pan Y, et al. Do tumor cavitation and sex in resected stage I non-small-cell lung cancer correlate with prognosis?. World J Surg, 2009, 33(3): 497-504. doi: 10.1007/s00268-008-9859-3 19115028

[25] Nguyen NC, Abhishek K, Nyon S, et al. Are there radiographic, metabolic, and prognostic differences between cavitary and noncavitary nonsmall cell lung carcinoma? A retrospective fluorodeoxyglucose positron emission tomography/computed tomography study. Ann Thorac Med, 2016, 11(1): 49-54. doi: 10.4103/1817-1737.165296 26933457 PMC4748615

[26] Suzuki K, Watanabe S, Wakabayashi M, et al. A nonrandomized confirmatory phase III study of sublobar surgical resection for peripheral ground glass opacity dominant lung cancer defined with thoracic thin-section computed tomography (JCOG0804/WJOG4507L). J Clin Oncol, 2017, 35(15_suppl): 8561. doi: 10.1200/JCO.2017.35.15_suppl.8561

[27] Saji H, Okada M, Tsuboi M, et al. Segmentectomy versus lobectomy in small-sized peripheral non-small-cell lung cancer (JCOG0802/WJOG4607L): a multicentre, open-label, phase 3, randomised, controlled, non-inferiority trial. Lancet, 2022, 399(10335): 1607-1617. doi: 10.1016/S0140-6736(21)02333-3 35461558

[28] Suzuki K. JCOG0201 defined “radiological early peripheral lung adenocarcinoma”. J Thorac Oncol, 2011, 6(8): 1452-1453. doi: 10.1097/JTO.0b013e318223c415 21847069

[29] Aokage K, Suzuki K, Saji H, et al. Segmentectomy for ground-glass-dominant lung cancer with a tumour diameter of 3 cm or less including ground-glass opacity (JCOG1211): a multicentre, single-arm, confirmatory, phase 3 trial. Lancet Respir Med, 2023, 11(6): 540-549. doi: 10.1016/S2213-2600(23)00041-3 36893780

[30] Wang C, Wu Y, Li J, et al. Distinct clinicopathologic factors and prognosis based on the presence of ground-glass opacity components in patients with resected stage I non-small cell lung cancer. Ann Transl Med, 2020, 8(18): 1133. doi: 10.21037/atm-20-4971 33240982 PMC7576059

[31] Park S, Lee SM, Choe J, et al. Recurrence patterns and patient outcomes in resected lung adenocarcinoma differ according to ground-glass opacity at CT. Radiology, 2023, 307(3): e222422. doi: 10.1148/radiol.222422 36943079

[32] Sun F, Huang Y, Yang X, et al. Solid component ratio influences prognosis of GGO-featured IA stage invasive lung adenocarcinoma. Cancer Imaging, 2020, 20(1): 87. doi: 10.1186/s40644-020-00363-6 33308323 PMC7733294

[33] Covington MF, Koppula BR, Fine GC, et al. PET-CT in clinical adult oncology: II. Primary thoracic and breast malignancies. Cancers, 2022, 14(11): 2689. doi: 10.3390/cancers14112689 35681669 PMC9179296

[34] Monaco L, Gemelli M, Gotuzzo I, et al. Metabolic parameters as biomarkers of response to immunotherapy and prognosis in non-small cell lung cancer (NSCLC): A real world experience. Cancers, 2021, 13(7): 1634. doi: 10.3390/cancers13071634 33915801 PMC8037395

[35] Zhang H, Wroblewski K, Liao S, et al. Prognostic value of metabolic tumor burden from (18)F-FDG PET in surgical patients with non-small-cell lung cancer. Acad Radiol, 2013, 20(1): 32-40. doi: 10.1016/j.acra.2012.07.002 22999369

[36] Liu J, Dong M, Sun X, et al. Prognostic value of ^18^F-FDG PET/CT in surgical non-small cell lung cancer: a meta-analysis. PLoS One, 2016, 11(1): e0146195. doi: 10.1371/journal.pone.0146195 26727114 PMC4699812

[37] Sharma A, Mohan A, Bhalla AS, et al. Role of various metabolic parameters derived from baseline ^18^F-FDG PET/CT as prognostic markers in non-small cell lung cancer patients undergoing platinum-based chemotherapy. Clin Nucl Med, 2018, 43(1): e8-e17. doi: 10.1097/RLU.0000000000001886 29112011

[38] Dai C, Shen J, Ren Y, et al. Choice of surgical procedure for patients with non-small-cell lung cancer ≤ 1 cm or > 1 to 2 cm among lobectomy, segmentectomy, and wedge resection: a population-based study. J Clin Oncol, 2016, 34(26): 3175-3182. doi: 10.1200/JCO.2015.64.6729 27382092

[39] Widder J, Van De Wauwer C, Langendijk JA. Lobectomy or sublobectomy for small non-small-cell lung cancer: the question remains. J Clin Oncol, 2017, 35(5): 572-573. doi: 10.1200/JCO.2016.70.0872 27893336

[40] Ma H, Cheng J, Yu Y, et al. Adjuvant treatment can improve prognosis in patients with non-small cell lung cancer ≤3 cm after sublobectomy: a propensity score analysis. J Thorac Dis, 2021, 13(1): 312-321. doi: 10.21037/jtd-20-3448 33569211 PMC7867827

[41] Bepler G, Olaussen KA, Vataire AL, et al. ERCC1 and RRM1 in the international adjuvant lung trial by automated quantitative in situ analysis. Am J Pathol, 2011, 178(1): 69-78. doi: 10.1016/j.ajpath.2010.11.029 21224045 PMC3069904

[42] Rosell R, Skrzypski M, Jassem E, et al. BRCA1: a novel prognostic factor in resected non-small-cell lung cancer. PLoS One, 2007, 2(11): e1129. doi: 10.1371/journal.pone.0001129 17987116 PMC2042516

[43] Tsao MS, Aviel-Ronen S, Ding K, et al. Prognostic and predictive importance of p53 and RAS for adjuvant chemotherapy in non small-cell lung cancer. J Clin Oncol, 2007, 25(33): 5240-5247. doi: 10.1200/JCO.2007.12.6953 18024870

[44] Qiu B, Guo W, Zhang F, et al. Dynamic recurrence risk and adjuvant chemotherapy benefit prediction by ctDNA in resected NSCLC. Nat Commun, 2021, 12(1): 6770. doi: 10.1038/s41467-021-27022-z 34799585 PMC8605017

[45] Song J, Yan Y, Chen C, et al. Tumor mutational burden and efficacy of chemotherapy in lung cancer. Clin Transl Oncol, 2023, 25(1): 173-184. doi: 10.1007/s12094-022-02924-6 35995891

[46] Yang SR, Schultheis AM, Yu H, et al. Precision medicine in non-small cell lung cancer: Current applications and future directions. Semin Cancer Biol, 2022, 84: 184-198. doi: 10.1016/j.semcancer.2020.07.009 32730814

[47] Beasley MB, Milton DT. ASCO provisional clinical opinion: epidermal growth factor receptor mutation testing in practice. J Oncol Pract, 2011, 7(3): 202-204. doi: 10.1200/JOP.2010.000166 21886505 PMC3092665

[48] Kwak EL, Bang YJ, Camidge DR, et al. Anaplastic lymphoma kinase inhibition in non-small-cell lung cancer. N Engl J Med, 2010, 363(18): 1693-1703. doi: 10.1056/NEJMoa1006448 20979469 PMC3014291

[49] Lindeman NI, Cagle PT, Aisner DL, et al. Updated molecular testing guideline for the selection of lung cancer patients for treatment with targeted tyrosine kinase inhibitors: Guideline from the College of American Pathologists, the International Association for the Study of Lung Cancer, and the Association for Molecular Pathology. J Thorac Oncol, 2018, 13(3): 323-358. doi: 10.1016/j.jtho.2017.12.001 29396253

[50] Murayama T, Nakaoku T, Enari M, et al. Oncogenic fusion gene CD74-NRG1 confers cancer stem cell-like properties in lung cancer through a IGF2 autocrine/paracrine circuit. Cancer Res, 2016, 76(4): 974-983. doi: 10.1158/0008-5472.CAN-15-2135 26837769

[51] Dhanasekaran SM, Balbin OA, Chen G, et al. Transcriptome meta-analysis of lung cancer reveals recurrent aberrations in NRG1 and Hippo pathway genes. Nat Commun, 2014, 5: 5893. doi: 10.1038/ncomms6893 25531467 PMC4274748

[52] Gay ND, Wang Y, Beadling C, et al. Durable response to afatinib in lung adenocarcinoma harboring NRG1 gene fusions. J Thorac Oncol, 2017, 12(8): e107-e110. doi: 10.1016/j.jtho.2017.04.025 28502724

[53] Cheema PK, Doherty M, Tsao MS. A case of invasive mucinous pulmonary adenocarcinoma with a CD74-NRG1 fusion protein targeted with afatinib. J Thorac Oncol, 2017, 12(12): e200-e202. doi: 10.1016/j.jtho.2017.07.033 29169524

[54] Drilon A, Somwar R, Mangatt BP, et al. Response to ERBB3-directed targeted therapy in NRG1-rearranged cancers. Cancer Discov, 2018, 8(6): 686-695. doi: 10.1158/2159-8290.CD-17-1004 29610121 PMC5984717

[55] Lanman BA, Allen JR, Allen JG, et al. Discovery of a covalent inhibitor of KRAS(G12C) (AMG 510) for the treatment of solid tumors. J Med Chem, 2020, 63(1): 52-65. doi: 10.1021/acs.jmedchem.9b01180 31820981

[56] Hallin J, Engstrom LD, Hargis L, et al. The KRAS (G12C) inhibitor MRTX849 provides insight toward therapeutic susceptibility of KRAS-mutant cancers in mouse models and patients. Cancer Discov, 2020, 10(1): 54-71. doi: 10.1158/2159-8290.CD-19-1167 31658955 PMC6954325

[57] Layfield LJ, Hammer RD, White SK, et al. Molecular testing strategies for pulmonary adenocarcinoma: an optimal approach with cost analysis. Arch Pathol Lab Med, 2019, 143(5): 628-633. doi: 10.5858/arpa.2018-0218-OA 30576239

[58] Gregory JR, Douglas EW, David SE, et al. Non-small cell lung cancer, version 4. 2024, NCCN Clinical Practice Guidelines in Oncology. J Natl Compr Canc Netw, 2024, 22(4): 249-274. doi: 10.6004/jnccn.2204.0023 38754467

[59] Park SY, Lee JG, Kim J, et al. Efficacy of platinum-based adjuvant chemotherapy in T2aN0 stage IB non-small cell lung cancer. J Cardiothorac Surg, 2013, 8: 151. doi: 10.1186/1749-8090-8-151 23759129 PMC3684513

[60] Morgensztern D, Du L, Waqar SN, et al. Adjuvant chemotherapy for patients with T2N0M0 NSCLC. J Thorac Oncol, 2016, 11(10): 1729-1735. doi: 10.1016/j.jtho.2016.05.022 27287414 PMC5141602

[61] Tu Z, Tian T, Chen Q, et al. Overall survival analyses following adjuvant chemotherapy or nonadjuvant chemotherapy in patients with stage IB non-small-cell lung cancer. J Oncol, 2021, 2021: 8052752. doi: 10.1155/2021/8052752 34335761 PMC8313364

[62] Xu Y, Wan B, Zhu S, et al. Effect of adjuvant chemotherapy on survival of patients with 8^th^ edition stage IB non-small cell lung cancer. Front Oncol, 2021, 11: 784289. doi: 10.3389/fonc.2021.784289 35155190 PMC8828472

[63] Zhang P, Duan J, Bai H, et al. Influence of adjuvant chemotherapy on survival for patients with stage IB and IIA non-small cell lung cancer. Thorac Cancer, 2021, 12(1): 30-39. doi: 10.1111/1759-7714.13685 33111432 PMC7779205

[64] Cheng YF, Chen YL, Liu CC, et al. Adjuvant chemotherapy in pathological node-negative non-small cell lung cancer. Sci Rep, 2023, 13(1): 19137. doi: 10.1038/s41598-023-46679-8 37932436 PMC10628181

[65] Wang J, Wu N, Lv C, et al. Should patients with stage IB non-small cell lung cancer receive adjuvant chemotherapy? A comparison of survival between the 8^th^ and 7^th^ editions of the AJCC TNM staging system for stage IB patients. J Cancer Res Clin Oncol, 2019, 145(2): 463-469. doi: 10.1007/s00432-018-2801-7 30474757 PMC11810431

[66] Shen ZQ, Feng KP, Fang ZY, et al. Influence of adjuvant chemotherapy on survival for patients with completely resected high-risk stage IB NSCLC. J Cardiothorac Surg, 2024, 19(1): 1. doi: 10.1186/s13019-023-02457-1 38166960 PMC10763355

[67] Choi J, Oh JY, Lee YS, et al. Clinical efficacy of adjuvant chemotherapy in stage IB (< 4 cm) non-small cell lung cancer patients with high-risk factors. Korean J Intern Med, 2022, 37(1): 127-136. doi: 10.3904/kjim.2020.011 32872735 PMC8747921

[68] Wightman SC, Lee JY, Ding L, et al. Adjuvant chemotherapy for visceral pleural invasion in 3-4-cm non-small-cell lung cancer improves survival. Eur J Cardiothorac Surg, 2022, 62(1): ezab498. doi: 10.1093/ejcts/ezab498 35325098

[69] Tsutani Y, Imai K, Ito H, et al. Adjuvant chemotherapy for high-risk pathologic stage I non-small cell lung cancer. Ann Thorac Surg, 2022, 113(5): 1608-1616. doi: 10.1016/j.athoracsur.2021.04.108 34186090

[70] Arriagada R, Auperin A, Burdett S, et al. Adjuvant chemotherapy, with or without postoperative radiotherapy, in operable non-small-cell lung cancer: two meta-analyses of individual patient data. Lancet, 2010, 375(9722): 1267-1277. doi: 10.1016/S0140-6736(10)60059-1 20338627 PMC2853682

[71] Tsutani Y, Goldman J W, Dacic S, et al. Adjuvant osimertinib vs. placebo in completely resected stage IA2-IA3 EGFR-mutated NSCLC: ADAURA2. Clin Lung Cancer, 2023, 24(4): 376-380. doi: 10.1016/j.cllc.2023.02.002 36872181

[72] Zhao K, Yang L, Liu L, et al. Real-world efficacy of adjuvant therapy for totally resected stage I lung adenocarcinoma patients with pathological high-risk factors: propensity score analysis. BMC Surg, 2024, 24(1): 140. doi: 10.1186/s12893-024-02428-w 38720305 PMC11080149

[73] Ou W, Li N, Wang BX, et al. Adjuvant icotinib versus observation in patients with completely resected EGFR-mutated stage IB NSCLC (GASTO1003, CORIN): a randomised, open-label, phase 2 trial. EClinicalMedicine, 2023, 57: 101839. doi: 10.1016/j.eclinm.2023.101839 36816343 PMC9932314

[74] Solomon B, Ahn J, Dziadziuszko R, et al. LBA2 ALINA: Efficacy and safety of adjuvant alectinib versus chemotherapy in patients with early-stage ALK+ non-small cell lung cancer (NSCLC). Ann Oncol, 2023, 34(supplement 2): S1295-S1296. doi: 10.1016/j.annonc.2023.10.051

[75] Govindan R, Mandrekar SJ, Gerber DE, et al. ALCHEMIST trials: A golden opportunity to transform outcomes in early-stage non-small cell lung cancer. Clin Cancer Res, 2015, 21(24): 5439-5444. doi: 10.1158/1078-0432.CCR-15-0354 26672084 PMC4683399

[76] Subbiah V, Velcheti V, Tuch BB, et al. Selective RET kinase inhibition for patients with RET-altered cancers. Ann Oncol, 2018, 29(8): 1869-1876. doi: 10.1093/annonc/mdy137 29912274 PMC6096733

[77] Tsuboi M, Goldman JW, Wu YL, et al. LIBRETTO-432, a phase III study of adjuvant selpercatinib or placebo in stage IB-IIIA RET fusion-positive non-small-cell lung cancer. Future Oncol, 2022, 18(28): 3133-3141. doi: 10.2217/fon-2022-0656 35950566

